# Exercise and the Microbiome: Mechanistic Perspectives of the Impact of Exercise on the Gut-Vascular Axis

**DOI:** 10.1128/mSystems.00650-21

**Published:** 2021-08-17

**Authors:** Marc D. Cook, Taylor Hogue

**Affiliations:** a North Carolina Agriculture and Technology State University, Department of Kinesiology, Greensboro, North Carolina, USA

**Keywords:** colon mucosa biome, dysbiosis, exercise, gut microbiome, hypertension

## Abstract

Given the participation of the microbiome in human health and disease, understanding the context of host-microbe interactions involved in vascular pathophysiology is now evolving through identifying microbial communities, specific taxa, and metabolic profiling which can be coupled to human health outcomes. Exercise has been used to define mechanisms related to improved vascular health, which may involve the microbiome. Motivated by the clinical significance that both exercise and the gut microbiome have; the objective of our work is to assist in defining the gut-vascular axis while identifying biomarkers of gut microbial health linked to vascular function. In this commentary, we will provide context to the mechanistic perspectives of exercise-induced improvements in gut microbial characteristics coupled to vascular health outcomes and offer insight on necessary future prospective investigations.

## COMMENTARY

We now have an understanding that the gut microbiome, specifically gut dysbiosis (a poorly diverse and unhealthy gut microbial profile), has strong and consistent associations with host health and disease. In the context of vascular health, products of microbial metabolism have been shown to promote processes that increase cardiovascular disease (CVD) risk (1, 2). Emerging host-microbial interaction data have begun to define key metabolic processes in the gut correlated with increased CVD risk in animal models and humans (3). Interventions targeting the gut microbiome may have promise in reducing risks; however, human intervention studies and mechanistic studies at the cellular level to define mechanisms of action are rare. Exercise, a lifestyle intervention that independently reduces CVD risk factors (4) and increases gut microbial diversity and short-chain fatty acid (SCFA) production (5), has presented a unique opportunity for our laboratory to vigorously work toward updating the mechanistic perspective related to the impact of exercise on gut microbial SCFA production coupled to endothelial/vascular health and hypertension (HTN) (i.e., gut-vascular axis). Interestingly in athletes, HTN is the most common CVD risk factor (6). Therefore, our work in defining gut microbial dynamics and SCFA biomarkers includes athletic populations, sedentary individuals, and exercise interventions (supervised exercise training) to understand distinct microbial characteristics. Lastly, endothelial cells (EC), which line the blood vessels and control blood pressure (BP), are responsive to beneficial gut-derived metabolites (SCFAs) that can improve BP (7). Our lab uses an innovative tool of an *in vitro* exercise mimetic (i.e., laminar shear stress [LSS]) (8) to map signaling pathways in cultured EC that may be similarly and differentially related to the independent beneficial effects of exercise and gut-derived SCFAs on EC function. With this, our work will expand our understanding of exercise-induced gut microbial SCFA-producing capacity and the SCFAs’ relationship with EC function and BP.

**FIG 1:**
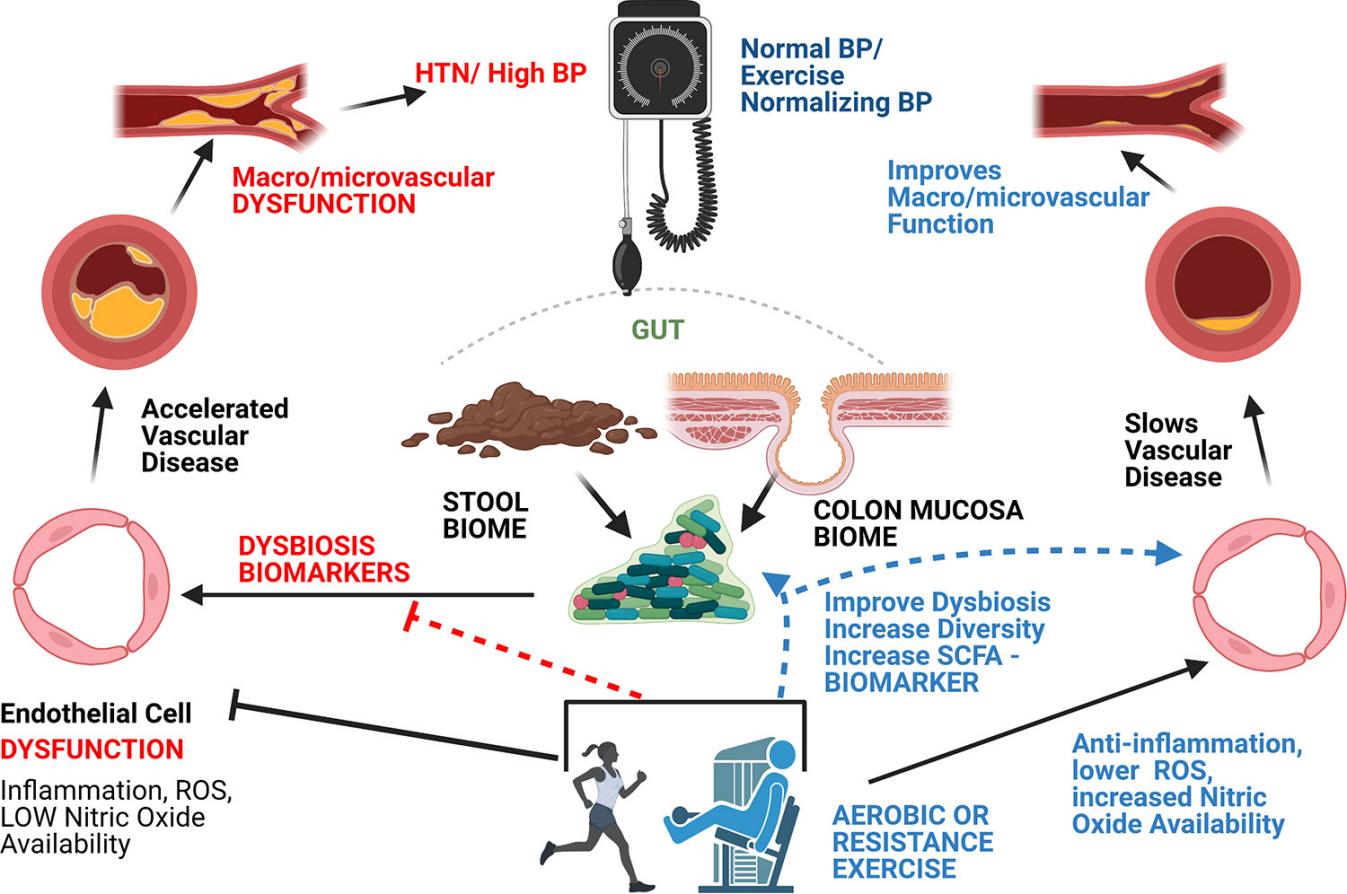
Overview. Our work seeks to define how exercise attenuates gut dysbiosis and identify biomarkers related to vascular health. We hypothesize that exercise promotes functional shifts to attenuate dysbiosis, increase gut SCFA production, improve EC dysfunction via increasing circulation of SCFAs, and normalize BP in individuals with HTN. ROS, reactive oxygen species.

The scope of our work is to fill the gap in knowledge concerning the role of exercise in CVD risk and the interactions within the gut-vascular axis by (i) comparing and contrasting human gut microbial profiles between exercise-trained (e.g., athletes) and sedentary individuals; (ii) influencing beneficial gut microbiome adaptations, via supervised exercise training, in individuals with and without HTN to identify functional shifts in metabolic capacity and specific microbial taxa associated with exercise-induced improvements in BP; and (iii) utilizing an *in vitro* exercise mimetic (LSS) in cultured EC to define the molecular mechanisms which protect and/or improve endothelial health stimulated by both exercise and biomarkers of gut microbial metabolism (i.e., SCFAs) (Fig. 1 shows overview).

## A. VASCULAR HEALTH AND MICROBIOME

The rising trend of specific risk factors that can make one prone to CVD, including obesity, high BP, and lack of exercise, obliges the quest for defining their relationship with the gut microbiome and identifying more efficient approaches to preventing and altering the trajectory of these disorders (1). Studies have shown when the diversity and structure of the gut microbiota and its related metabolites are altered, it may affect pathogenesis and development in diseases including vascular disease, such as HTN, heart failure, and stroke (9). The most modifiable CVD risk factor is HTN, and the ECs that line the blood vessels have a significant role in BP control and are responsive to gut microbial metabolites, such as SCFAs. SCFAs, such as butyrate, are products of microbial fermentation in the colon that are absorbed into the systemic circulation. As we are interested in the stool microbiome, we are carefully investigating the colon mucosal microbiome also. We believe the colon mucosal microbiome depot may have a significant impact on overall health given its spatial and functional relationship with the distal colon tissue. Our interest in the colon mucosal microbiome started when we discovered that our exercise interventions in mouse models of ulcerative colitis lowered colon inflammatory burden that was associated with reduced colon mucosal microbial biomass with a significant increase in beneficial (anti-inflammatory) microbes in the colon mucosa samples (10). We are currently analyzing human colon mucosal samples collected during clinical procedures (e.g., unprepped flexible sigmoidoscopies) to compare the stool and colon mucosal biomes.

Unpublished data in a previous study informed us that there may also be a significant vascular component to gut inflammation (11) and initiated exploration into EC dysfunction. With that, unpublished preliminary *in vitro* data from our lab informed us that butyrate has anti-inflammatory properties on EC and can attenuate EC dysfunction. Currently, we are working to define SCFA signaling pathways related to inflammatory processes in EC function. Additionally, we are utilizing our *in vitro* exercise mimetic in cultured EC to determine distinct signaling pathways associated with SCFA and our exercise stimulation on EC inflammatory profiles. In the near future, we expect to define how SCFAs, the microbes that produce them, and exercise may superimpose their effects to improve endothelial function to mitigate HTN.

## B. EXERCISE AND VASCULAR HEALTH AND GUT MICROBIOME

Concerning exercise, our work assesses how exercise improves vascular health (12). Again, our initial introduction to the role of exercise in gut microbial characteristics came when we reported that exercise stimulated functional shifts in the gut microbiome of exercise-trained mice in a model of gut inflammation (10, 11). More recently, our human intervention work has shown that exercise, independent of diet, stimulates changes in the gut microbiome that are thought to be related to improved health (e.g., increasing SCFA-producing capacity), where changes are transient if one discontinues exercise (13). While the direct mechanisms associated with exercise-induced changes in gut microbial composition are not understood, we are working to identify functional shifts in microbial taxa (through 16S rRNA and whole-genome sequencing) and quantifying circulating SCFAs (acetate, propionate, and butyrate) in the gut and blood to identify fundamental pathways of the gut-vascular axis. Our goal is to establish an experimental rigor that will stabilize our capacity to consistently identify key biomarkers of gut dysbiosis and provide insight into microbial metabolic profiles associated with CV health and CVD risk status. Ultimately, we expect our work to discover mechanistic perspectives related to exercise-induced improvements in HTN and CVD risk through its role in improving gut microbial SCFA-producing capacity and circulating SCFAs to promote vascular anti-inflammation. Importantly, we are including exercise-trained individuals (e.g., athletic populations) and supervised exercise training interventions to execute gut microbial metabolic profiling and identification of specific microbial taxa associated with shifts in BP. Gut microbial composition is significantly different in athletes compared to sedentary individuals (14), yet both experience HTN. The goal, with our approach in identifying specific taxa in both human stool and colon mucosa, is to expand our understanding of gut microbial characteristics in sedentary and active individuals while identifying novel quantifiable biomarkers of gut metabolic profiles to define their impact on BP.

## INFLUENCE ON THE FIELD

Defining the impact of exercise-induced microbial alterations on vascular health versus canonical exercise-induced vascular adaptations is a significant challenge in this field. Our lab’s approach is to work to isolate mechanistic avenues that link exercise-induced gut-derived SCFAs to endothelial function and the gut-vascular axis. Future studies must include germfree mouse cecal/fecal transplant studies in HTN pathology and isolated vessel studies. We are realizing that our work, utilizing exercise as a novel tool to elicit shifts in microbial metabolic profiles in mild to moderate HTN, has significant potential in revealing functional shifts of the gut microbiome linked to improved vascular health and potentially other chronic diseases. Our work in identifying gut metabolic profiles (specific taxa and circulating biomarkers) of gut microbial health is essential to developing strategies in predicting and reducing CVD risk while advancing therapies to improve gut microbial health.
